# Quantitative Dynamic Contrast-Enhanced Magnetic Resonance Imaging (DCE-MRI) in Hepatocellular Carcinoma: A Review of Emerging Applications for Locoregional Therapy

**DOI:** 10.3390/bioengineering12080870

**Published:** 2025-08-12

**Authors:** Xinyi M. Li, Tu Nguyen, Hiro D. Sparks, Kyunghyun Sung, Jason Chiang

**Affiliations:** Department of Radiological Sciences, University of California, Los Angeles, CA 92521, USA; xinyli@mednet.ucla.edu (X.M.L.); travisnguyen@mednet.ucla.edu (T.N.); hdsparks@mednet.ucla.edu (H.D.S.); ksung@mednet.ucla.edu (K.S.)

**Keywords:** hepatocellular carcinoma (HCC), dynamic contrast-enhanced MRI (DCE-MRI), locoregional therapy, tumor perfusion, imaging biomarker, thermal ablation, transarterial chemoembolization (TACE), transarterial radioembolization (TARE)

## Abstract

Quantitative dynamic contrast-enhanced magnetic resonance imaging (DCE-MRI) is emerging as a valuable tool for assessing tumor and parenchymal perfusion in the liver, playing a developing role in locoregional therapies (LRTs) for hepatocellular carcinoma (HCC). This review explores the conceptual underpinnings and early investigational stages of DCE-MRI for LRTs, including thermal ablation, transarterial chemoembolization (TACE), and transarterial radioembolization (TARE). Preclinical and early-phase studies suggest that DCE-MRI may offer valuable insights into HCC tumor microvasculature, treatment response, and therapy planning. In thermal ablation therapies, DCE-MRI provides a quantitative measurement of tumor microvasculature and perfusion, which can guide more effective energy delivery and estimation of ablation margins. For TACE, DCE-MRI parameters are proving their potential to describe treatment efficacy and predict recurrence, especially when combined with adjuvant therapies. In ^90^Y TARE, DCE-MRI shows promise for refining dosimetry planning by mapping tumor blood flow to improve microsphere distribution. However, despite these promising applications, there remains a profound gap between early investigational studies and clinical translation. Current quantitative DCE-MRI research is largely confined to phantom models and initial feasibility assessments, with robust retrospective data notably lacking and prospective clinical trials yet to be initiated. With continued development, DCE-MRI has the potential to personalize LRT treatment approaches and serve as an important tool to enhance patient outcomes for HCC.

## 1. Introduction

Hepatocellular carcinoma (HCC) is the most prevalent form of primary liver malignancy, ranking as the third leading cause of cancer deaths globally, with a rapidly growing rate of incidence [1]. The treatment of HCC is broadly guided by the Barcelona Clinic Liver Cancer (BCLC) criteria [2], which stratify patients to either a combination of surgical, locoregional, or systemic therapies, depending on the stage of the disease, underlying liver function, and the functional status of the patient. For patients with early-stage HCC, liver transplantation represents the ideal curative approach, with surgical resection serving as another option. However, a shortage of donor allografts restricts transplantation, and many patients are ineligible for surgical resection due to factors such as tumor burden, location, or compromised underlying liver function [3]. In this context, locoregional treatments such as radiofrequency ablation (RFA), microwave ablation (MWA), transarterial chemoembolization (TACE), and transarterial radioembolization (TARE) have become increasingly recognized as alternatives for patients ineligible for surgery or as bridges to prevent further tumor growth for patients listed for liver transplant [2]. However, the efficacy of these interventions is highly dependent on the precise targeting and adequate assessment of the tumor’s vascular characteristics [4]. Consequently, imaging can play a vital role in this context, helping not only with diagnosing HCC but also with staging and optimizing treatment strategies [5]. While conventional imaging modalities like multiphasic contrast-enhanced MRI (CE-MRI) are fundamental for HCC diagnosis, they often provide limited insight into the functional heterogeneity of tumor perfusion and the tumor microenvironment. This limitation can impede optimal locoregional therapy (LRT) planning and the early prediction of treatment response.

Perfusion magnetic resonance imaging (MRI) is emerging as an increasingly investigated technique for evaluating and managing HCC, allowing physicians to assess underlying liver blood flow and microcirculation. Historically, these parameters were not adopted due to both the resolution limitations of MRI and the uncertain clinical value of quantitative perfusion metrics [5,6]. Computed tomography perfusion (CTP) provides high spatial and temporal resolution as well as straightforward quantification of contrast concentration, as attenuation is directly proportional to concentration. However, CTP also exposes patients to ionizing radiation, which can be undesirable with repeated imaging. In contrast, MRI avoids the risks of ionizing radiation altogether, allowing for a higher number of protocols to be run without risking excess radiation exposure [5]. Within MRI, perfusion can be assessed using methods such as arterial spin labeling (ASL), dynamic susceptibility contrast (DSC) MR perfusion, or dynamic contrast-enhanced MRI (DCE-MRI). The scope of this review will focus on DCE-MRI, given its widespread investigation for quantitative tissue characterization in liver imaging [5].

For the liver, traditional methods for characterizing focal lesions primarily rely on visual assessment of contrast enhancement patterns on three-phased CE-MRI scans. These patterns arise from the distribution of contrast agent within the liver over time, a process influenced by underlying microcirculatory characteristics [7]. By rapidly measuring tissue enhancement, DCE-MRI provides a quantitative extension to traditional visual interpretations of enhancement parameters. It enables the measurement of tissue perfusion parameters such as blood flow, blood volume, and capillary permeability, which can help to predict the behavior of the tumor and guide therapeutic decision-making of different treatment modalities [8].

This review aims to explore the integration of DCE-MRI with locoregional therapies for HCC. It is important to note that while the BCLC framework guides overall treatment strategy, advanced imaging techniques like quantitative DCE-MRI remain primarily investigational in this context and are not currently incorporated for specific decision-making within locoregional therapy pathways in these or other major clinical guidelines [2]. We will investigate the principles of MR perfusion using DCE-MRI and examine its applications across various locoregional therapies. Specifically, we will discuss how insights into underlying tumor vascularity gained from DCE-MRI may help to optimize thermal ablation therapies such as RFA and MWA. We will also cover its application in TACE, where DCE-MRI can aid in assessing tumor perfusion and response after treatment. Finally, we will explore how DCE-MRI has the potential to improve dosimetry for ^90^Y TARE. By reviewing the latest research, we highlight the growing utility of DCE-MRI for optimizing locoregional therapies in HCC. With its ability to provide quantitative data beyond standard visual assessment of tumor perfusion, DCE-MRI shows promise in guiding therapeutic decisions and improving patient outcomes in HCC patients.

## 2. Principles of Dynamic Contrast-Enhanced MRI (DCE-MRI)

**Figure 1:**
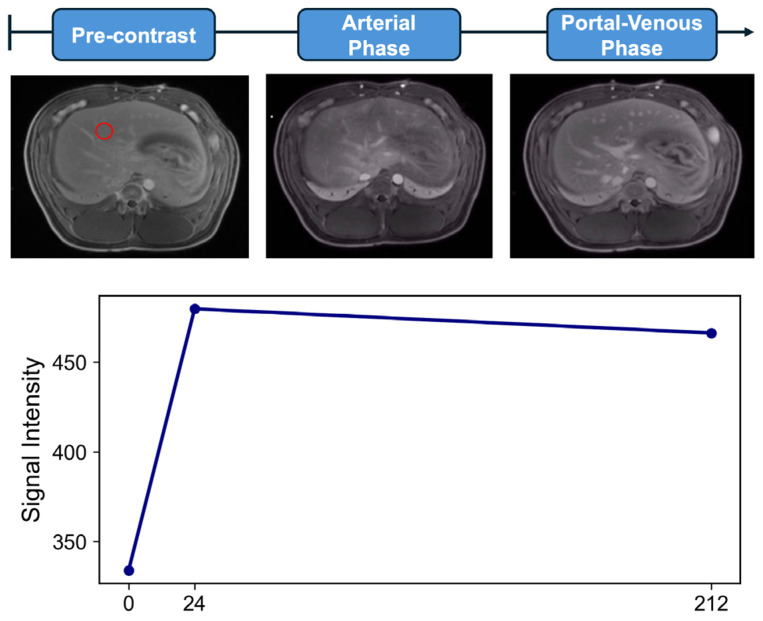
**Conventional multi-phase contrast-enhanced MRI (CE-MRI) in a porcine model.** This technique involves acquiring images at distinct, specific time points after contrast administration, typically corresponding to key vascular phases such as the pre-contrast, arterial, and portal venous phases shown. The red circle in the (**top panel**) indicates the voxel from which the signal intensity is measured to generate the curve in the bottom panel. The resulting signal intensity versus time curve (**bottom panel**) is sparsely sampled, given the small number of time points. Note the persistent aortic enhancement in the portal venous phase, reflecting the rapid arterial transit times observed in in vivo porcine models. All animal procedures were approved by the Institutional Animal Care and Use Committee (protocol ARC-2016-053) and performed in accordance with national guidelines.

**Figure 2:**
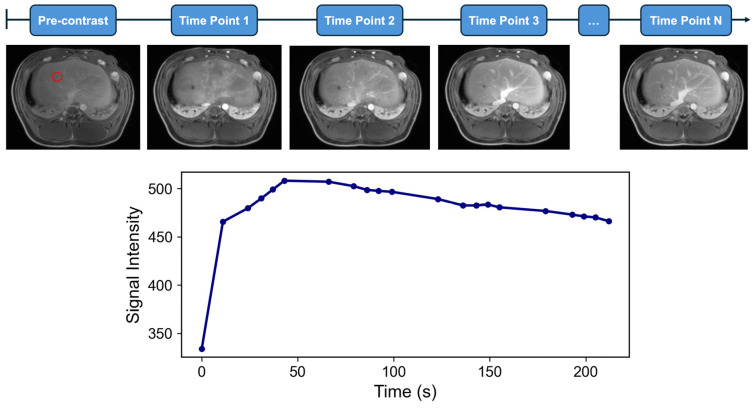
**Dynamic contrast-enhanced MRI (DCE-MRI) for quantitative analysis in a porcine model.** The red circle in the (**top panel**) indicates the voxel from which the signal intensity is measured. In contrast to conventional CE-MRI, DCE-MRI utilizes rapid, sequential image acquisition over numerous time points (1-N) following contrast injection in an in vivo porcine liver model. This high temporal resolution captures the dynamic passage of the contrast agent, yielding a densely sampled signal intensity versus time curve (**bottom panel**). Data with high temporal resolution are essential for quantitative analysis, including pharmacokinetic modeling of tissue perfusion. The successful adoption of DCE-MRI as a quantitative tool is dependent on acquiring data with high temporal resolution, as this is fundamental to the accuracy of pharmacokinetic modeling [12]. All animal procedures were approved by the Institutional Animal Care and Use Committee (protocol ARC-2016-053) and performed in accordance with national guidelines.

**Figure 3:**
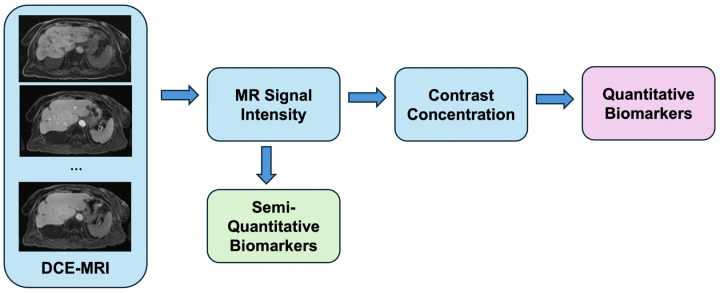
General processing pipeline for dynamic contrast-enhanced MRI (DCE-MRI). MR signal intensity derived from dynamic images can be used to generate semiquantitative biomarkers or converted to contrast agent concentration for deriving quantitative biomarkers through pharmacokinetic modeling.

MR contrast studies begin with the intravenous injection of a gadolinium-based contrast agent, which shortens the T1 relaxation time of tissues and enhances their signal intensity on T1-weighted images [9]. During this time, high-temporal-resolution imaging captures MR signal intensity (SI) as the contrast agent is perfused through tissue over time (Figure 1 and Figure 2). A 3D gradient echo sequence is widely used for liver DCE-MRI, with typical image acquisition parameters outlined in the Quantitative Imaging Biomarkers Alliance (QIBA) recommendations for DCE-MRI-derived biomarkers [10]. Hepatic contrast perfusion appears as an increasing enhancement on T1-weighted images. The reliable measurement of the pharmacokinetic (PK) perfusion parameters depends on accurately determining the gadolinium concentration ([Gd]) from the acquired SI for both the liver parenchyma and the vascular input [11]. Dynamic MRI data can be analyzed using either a model-free semiquantitative or a model-based quantitative approach (Table 1). The pipeline from acquiring dynamic signal data to deriving contrast concentration curves and ultimately applying these analytical approaches for semiquantitative and quantitative features is shown in Figure 3.

**Figure 4:**
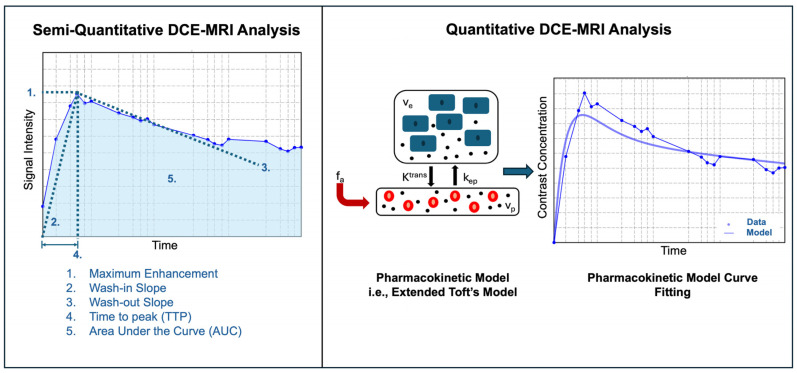
Diagram of semiquantitative and quantitative modeling approaches in DCE-MRI. The semiquantitative method examines the shape and features of the signal intensity curve to gain insights into underlying pathophysiology. Parameters such as the area under the curve (AUC), maximum enhancement, wash-in slope, wash-out slope, etc., can be derived from the signal intensity over time curve. The quantitative modeling graph highlights the process of tracer kinetic modeling, with a tracer kinetic model that fits a mathematical curve to the contrast concentration over time data. This modeling approach quantifies physiological parameters, such as the forward volume transfer constant (K^trans^) and the reverse volume transfer constant (k_ep_).

Model-free semiquantitative analysis involves examining the shape and features of the SI curves to gain insights into tumor pathophysiology (see Figure 4). Some key parameters derived from these curves include the area under the curve (AUC), which reflects the total volume flow over a specified time period; the peak signal intensity or peak enhancement ratio, which indicates the highest level of enhancement; the wash-in slope and wash-out slope, which measure the rate of flow; and the mean transit time (MTT), which represents the average time required for blood to traverse a tissue region. Although semiquantitative methods are widely investigated in the scientific literature due to their simplicity and lack of modeling requirements, they do not provide a direct assessment of physiological characteristics as quantitative models do [13].

Model-based quantitative analysis of DCE-MRI data involves deriving parameters that reflect the pharmacokinetics of the contrast agent. Following acquisition, signal intensities are converted into contrast agent concentrations over time, typically through a signal-to-concentration transformation that accounts for baseline T1 values and imaging parameters [14]. Next, the arterial input function (AIF) is selected either through direct measurement from a vascular structure such as the aorta or hepatic artery [15] or by estimation using a population-based model, which relies on averaged blood sample measurements from a cohort of subjects [16]. The AIF represents the time-varying concentration of contrast agent in the blood plasma and serves as the input for kinetic modeling. Tracer kinetic modeling then uses mathematical curve fitting to describe the tissue contrast concentration–time curves (see Figure 4), the details of which are beyond the scope of this review [17]. Most DCE-MRI quantitative analysis studies currently use in-house software. However, open-source packages for DCE-MRI model fitting are also available [18].

There are many physiologically relevant tracer compartment models that describe the contrast concentration–time curves. Each model has its own physiological justification, but there is no consensus on the most suitable model for DCE-MRI for the liver. The selection of each model relies on the specific context in which it is being used [9]. Tracer compartment models can be categorized into single-compartment models or multi-compartment models. Single-compartment models, such as the widely utilized standard Tofts model, assume the contrast agent is limited to a single space [19]. The standard Tofts model conceptualizes tissue as comprising blood plasma and one additional tissue compartment: the extravascular extracellular space (EES). It assumes that the contrast agent can move from the blood plasma into the EES and back again. This model is frequently employed due to its relative simplicity and its ability to estimate key microvascular parameters that describe this exchange. In this model, the tissue contrast concentration curve C_t_(t) is described as follows:

$$C_{t}\left(t\right)\;=\;K^{trans}\cdot \int_{0}^{t}C_{p}\left(\tau \right)\cdot e^{-k_{ep}(t-\tau )}d\tau$$

where K^trans^ is the volume transfer constant between plasma and EES, k_ep_ is the rate constant describing the reflux from EES to plasma, and C_p_(t) is the AIF. From these parameters, additional measures such as the extracellular volume fraction V_e_ (where V_e_ = K^trans^/k_ep_) can also be derived [19].

Alternatively, multi-compartment models such as the extended Tofts model [20] or the two-compartment exchange model [21] take into account the transit of contrast agent through both the vascular space and the extravascular extracellular space [13].

Since the liver is dual-supplied by the hepatic artery and portal vein, compartmental models can be further categorized into those that use a single arterial input function and those that incorporate both arterial and venous inputs [13].

**Figure 5:**
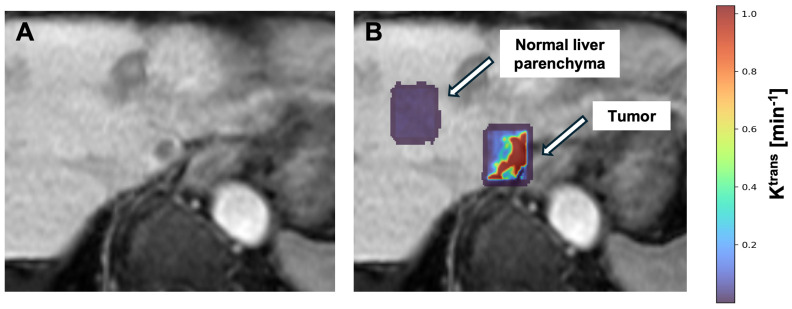
K^trans^ parametric map illustrating increased perfusion in a 10 mm LI-RADS 5 hepatocellular carcinoma (HCC) lesion (right ROI) in a 94-year-old patient compared to normal tissue parenchyma (left ROI). (**A**) Anatomical T1-weighted MRI obtained in the hepatobiliary phase. (**B**) K^trans^ map overlaid on the anatomical image, where warmer colors indicate higher K^trans^ values. These quantitative value maps have the potential to be leveraged to guide more accurate delivery of locoregional therapy.

Given these multiple degrees of freedom with the input, there are consequently many parameters that can be derived from mathematical models. Some of the key outputs of DCE-MRI are the forward and backward volume transfer constants, K^trans^ and k_ep_, respectively. These parameters reflect the movement of contrast agent between the bloodstream and the EES, offering valuable insights into microvascular blood flow, vessel permeability, and vascular density (Figure 5). Another important parameter is the volume fraction of EES (V_e_), which serves as an indirect measure of the tissue cellular density, further enhancing the understanding of the tissue microenvironment [13].

The aim in outlining these quantitative and semiquantitative post-processing methods is to provide the reader with a foundational understanding of the parameters relating to DCE-MRI referenced in key studies summarized in Table 2. A comprehensive literature review was performed to identify such studies using the electronic database PubMed. Search terms included combinations of “HCC”, “TACE”, “Ablation”, “Y90”, “DCE-MRI”, “Semi-quantitative”, “Quantitative”, “Ktrans”, “Kep”, and their variations. Studies were excluded if they involved conventional contrast-enhanced MRI, which is not to be confused with DCE-MRI (Figure 1 and Figure 2). A total of 12 unique studies were included in this review to represent the current scope of research applying quantitative DCE-MRI to locoregional therapies for HCC. Studies were selected based on the use of qualitative or quantitative DCE-MRI in the context of TACE, ablation, or Y^90^ therapy.

## 3. DCE-MRI in Ablation Therapy

**Figure 6:**
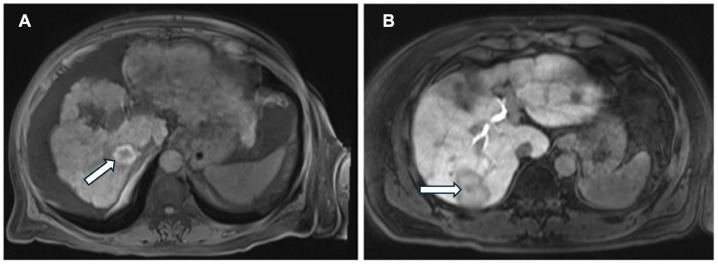
Variability in thermal ablation efficacy influenced by liver parenchymal differences, despite uniform energy delivery (65 W for 10 min). Arrows in the figure point towards the ablation zone. (**A**) A 2.5 cm ablation zone is shown on T1 non-contrast MRI with a clear margin surrounding the post-ablation tumor site. (**B**) In contrast, a 4.3 cm ablation zone is observed in a non-cirrhotic liver with HCC (hepatobiliary phase imaging). Given the same power and time, changes in ablation size and shape are likely related to surrounding blood vessel anatomy and underlying perfusional changes of the liver.

Image-guided percutaneous ablation is recognized as the therapy of choice for patients with early-stage HCC who are not candidates for surgery [2]. The goal of thermal ablation is to treat tumor tissue through the application of heat while minimizing thermal injury to nontarget organs [34]. The success of ablation therapy depends on accurately predicting the appropriate final ablation zone and margin, which is largely influenced by the power delivery and time. However, a key determinant of the final ablation zone is liver perfusion, which is influenced by underlying liver disease as well as the tumor’s vascular features (Figure 6) [23].

DCE-MRI has been explored for its potential to provide noninvasive, quantitative assessments of perfusion, which may help guide and evaluate ablation therapy. HCC tumor tissue can be heterogeneous with varying levels of internal perfusion [35]. Each focus of perfusion uniquely interacts with the thermal energy delivered by the ablation probe within the target zone. Areas with high vascularity may require higher amounts of energy to attain cytotoxic temperatures, and thus carry the risk of undertreatment and viable residual disease [36]. Conversely, areas with low vascularity may require less energy to adequately treat, with overtreatment potentially leading to inadvertent damage to nearby anatomy. Thus, it is critical to quantify the amount of perfusion that exists within the tumor and surrounding liver parenchyma. As such, mapping perfusion within and around the tumor could help inform and personalize ablation parameters. However, the clinical utility of quantitative DCE-MRI in this context remains largely investigational, lacking validation in large prospective trials, as demonstrated by the following studies.

Prior research has suggested that DCE-MRI may serve as a biomarker for assessing treatment response following thermal ablation [22,37]. In an early clinical study involving 50 patients, DCE was used to evaluate recurrent and residual HCC tumors following radiofrequency ablation, showing a decline in peri-ablated signal intensity over time [22]. Specifically, the proportion of patients with low T1 signal intensity in the peri-ablated zones on DCE-MRI decreased from 99% measured at 1 month to 11% at 4–6 months and only 7% after 9–12 months, respectively. This evolving signal pattern highlights DCE-MRI’s potential to detect subtle post-ablation tissue changes and to support long-term monitoring of tumor viability.

Supporting evidence from preclinical studies has further explored DCE-MRI’s potential to guide ablation therapy. In a porcine liver model, pharmacokinetic parameters K^trans^ and k_ep_ were used to quantify perfusion changes after embolization and showed a significant inverse correlation with ablation zone size, suggesting that regions with lower baseline perfusion might require less energy to achieve complete necrosis [38]. Furthermore, the relationship between quantitative DCE-MRI PK parameters and microvessel density was investigated in a rabbit liver tumor model following RFA. The study found significant positive correlations between the K^trans^ parameter and microvessel density in both the partially necrotic and viable tumor regions after radiofrequency ablation, indicating that K^trans^ values reflect microvascular characteristics of liver tumors [24]. These results support the potential of DCE-MRI in generating imaging biomarkers for predicting response to ablation therapy. Additionally, K^trans^ values have been found to be positively correlated with serum vascular endothelial growth factor (VEGF)—a protein regulating the growth of new blood vessels and vascular permeability [39]—levels in a rabbit liver cancer model under insufficient microwave ablation [25]. This observed correlation between VEGF and K^trans^ further implicates the biological relevance of K^trans^ measured by DCE-MRI in the context of thermal-ablation efficacy.

Overall, these findings reinforce the biological relevance of DCE-MRI biomarkers such as K^trans^ as physiologically meaningful indicators of tumor vascularity and predictors of thermal ablation efficacy. In addition, these studies point to the potential of DCE-MRI in enhancing the precision of ablation by tailoring energy delivery to tumor perfusion characteristics. Taken together, these insights highlight the growing potential of DCE-MRI to guide and refine clinical ablation strategies for HCC.

## 4. DCE-MRI in Transarterial Chemoembolization (TACE)

TACE is an established standard of care treatment option for patients with intermediate-stage HCC [40]. The procedure involves the infusion of chemo-embolic agents through selective catheterization directly into the hepatic artery branches supplying the tumor [40]. Established reasons to perform TACE include reducing tumor burden to meet transplant criteria, controlling tumor progression in patients awaiting transplantation, and prolonging survival in those who are not candidates for transplant [40]. The success of the procedure depends on accurate targeting of the tumor and effective embolization. Despite known survival advantages, the local recurrence rate following TACE remains high, resulting in many patients requiring multiple treatment sessions to achieve an adequate response [41]. This underscores the need for better predictive biomarkers and response assessment tools, a role for which quantitative DCE-MRI is being actively investigated.

This frequent need for retreatment highlights the importance of accurately assessing tumor response following TACE to avoid unnecessary procedures while detecting early residual or recurrent disease. The lipiodol used in TACE can be difficult to distinguish from true residual disease when using contrast-enhanced CT. Although direct comparisons between PK parameters and conventional imaging assessments remain lacking, early preclinical findings suggest these parameters may reflect underlying tumor biology more objectively by providing quantitative metrics that differentiate HCC from normal liver parenchyma [42]. As such, DCE-MRI with pharmacokinetic modeling remains a promising yet investigational tool for improving post-TACE response evaluation and guiding future treatment strategies.

Recent studies have demonstrated the clinical value of both qualitative and quantitative DCE-MRI features in this context. One strategy involves using DCE-MRI to identify imaging biomarkers predictive of TACE response in HCC patients. This study measured the ability of DCE-MRI to evaluate post-TACE treatment response in 30 patients. Markers such as the absence of arterial-phase hyperenhancement correctly identified non-viable tumors, and the presence of arterial-phase hyperenhancement reliably indicated viable tumors [26]. Beyond visual assessment, emerging artificial intelligence (AI) technologies, including radiomics and deep learning models, have also been increasingly explored for their potential to enhance response prediction and treatment monitoring in TACE. A recent study investigated this approach in a cohort of 175 patients, incorporating a novel spatial–temporal graph neural network model utilizing pre-TACE DCE-MRI data to predict treatment response [27]. This predictive model accurately classified tumors into viable, non-viable, or equivocal categories while also predicting growth patterns and perfusion changes on follow-up imaging. Radiomics-based models have further expanded the predictive utility of DCE-MRI. A multicenter study combined radiomic features from DCE-MRI with clinical variables to develop a logistic regression model that showed strong predictive ability for tumor response. This multicenter study identified 11 DCE-MRI radiomics features associated with tumor heterogeneity. In the training set, their radiomics model achieved an AUC of 0.83 for predicting tumor response, while a model based on clinical variables alone yielded an AUC of 0.77. These findings suggest that an MRI-based nomogram can effectively predict short-term response to TACE in HCC patients with tumors smaller than 5 cm [28]. Collectively, these findings highlight the feasibility of qualitative DCE-MRI to measure TACE outcomes.

In addition to imaging features, quantitative DCE-MRI parameters like distribution volume (DV) and K^trans^ have shown promise in evaluating treatment efficacy. A recent clinical study investigated the correlation between DV and K^trans^ of Gd-EOB-DTPA and sorafenib-combined TACE response in stage B or C HCC patients [30]. This study found that patients who responded to TACE exhibited a significant decrease in K^trans^ between pre-TACE and 3–10 days post-TACE. There was also a significant difference in DV between responders and non-responders at 3 and 10 days post-TACE. This study provides valuable insights into post-TACE angiogenesis, particularly changes in arterial and portal venous flows, which play a crucial role in disease progression and recurrence [42]. Moreover, DCE-MRI-derived semiquantitative metrics such as the time to peak parameter in subjects after TACE treatment have been shown to help differentiate post-TACE inflammation and necrosis from viable residual tumor, despite overlapping imaging features on conventional MRI [29]. These results validate the utility of DCE-MRI in quantifying the embolic effects of TACE and underscore the need for larger-scale studies to evaluate how such biomarkers predict tumor response and impact clinical decision-making.

Another study showed that the combination of TACE and the anti-angiogenic drug Sunitinib significantly reduced K^trans^ [31]. This reduction in K^trans^ implicated a positive tumor response to the treatment. The sensitivity of DCE-MRI to early anti-angiogenic effects has also been noted in studies of other liver tumors, such as colorectal cancer metastases. For instance, following treatment with agents like Bevacizumab, DCE-MRI can detect decreased K^trans^ and k_ep_ as early as one week post-anti-angiogenic treatment [43]. Furthermore, a decrease in these perfusion parameters compared to baseline was found to be correlated with longer survival outcomes and better response to Sunitinib treatment [31]. Overall, these findings highlight the potential of semiquantitative and quantitative biomarkers for predicting TACE response and guiding treatment decisions, including the combination of TACE with anti-angiogenic agents.

Collectively, these findings highlight the emerging potential of various DCE-MRI approaches, from qualitative visual assessment to quantitative AI-driven radiomics to quantitative biomarkers of embolic effects, in evaluating and predicting TACE outcomes. Advancements in artificial intelligence, including radiomics and deep learning models, particularly in the space of TACE response, may further enhance the predictive capabilities of DCE-MRI, allowing for more precise identification of patients who may benefit from TACE.

## 5. DCE-MRI in ^90^Y Mapping and Dosimetry

^90^Y TARE is a locoregional treatment modality historically performed primarily with palliative intent for patients with advanced disease or for those who failed to respond to other therapies [44]. Recent findings from the LEGACY (Local radioEmbolization using Glass Microspheres for the Assessment of Tumor Control with Y-90) study have demonstrated the efficacy of ^90^Y TARE using radiation segmentectomy in early-stage HCC [45]. ^90^Y TARE involves the injection of radioactive microspheres into the hepatic artery, which then lodge in the tumor’s microvasculature and deliver a localized radiation dose to the tumor tissue over time [44]. The success of this therapy is dependent on accurate dosimetry, which ensures that the tumor receives an adequate radiation dose while minimizing exposure to nontarget structures. Optimizing the tumor microsphere density has been proposed as a key factor in achieving favorable ^90^Y treatment outcomes [46]. In the DOSISPHERE-01 trial, study analysis demonstrated that personalized dosimetry significantly improved overall survival compared to standard dosimetry [47]. While the data are promising, heterogeneity in bead distribution within the tumor has not been well evaluated and is hypothesized to contribute to suboptimal rates of complete pathological necrosis [48]. This highlights the critical need for optimizing dose delivery based on individual tumor characteristics to enhance therapeutic efficacy.

When ordering a ^90^Y TARE dose, the operator is able to control two aspects of the therapy: the prescribed activity and the quantity of microspheres [49]. Achieving the optimal microsphere particle density in ^90^Y TARE is a delicate balance where, on one hand, a low particle density may lead to gaps between particles, diminishing the effective dose [50], while on the other hand, a high particle density could induce tumor hypoxia, rendering the tumors resistant to radiation [51]. Additionally, particles initially flow preferentially to the most vascularized regions of the tumor. Once these vessels are embolized, the particles then move to less vascular areas of the tumor and eventually to normal liver tissue, resulting in a dynamic distribution of microspheres as more are delivered [49]. Dosimetry for ^90^Y TARE relies on pre-treatment imaging, typically using Tc-99m macroaggregated albumin (MAA) SPECT scans, to simulate the distribution of ^90^Y microspheres. However, the MAA distribution often does not perfectly match the actual microsphere distribution, leading to discrepancies in the predicted versus actual radiation dose delivered to the tumor and surrounding tissues. Factors such as catheter positioning, tumor vascularity, and variations in blood flow can all affect the distribution of both MAA and ^90^Y microspheres [33]. These challenges underscore the importance of more precise imaging approaches that can improve dosimetry accuracy and better predict microsphere distribution, an area where DCE-MRI shows early, albeit investigational, promise.

DCE-MRI presents a promising tool for refining personalized dosimetry by providing quantitative insights into tumor perfusion and vascular heterogeneity. DCE-MRI could be used to refine dosimetry by mapping areas of high and low vascularity, thus guiding delivery to determine the optimal microsphere density and dose distribution [52]. Limited research has focused on optimizing microsphere distribution, a critical factor in the success of ^90^Y TARE therapy. While the clinical literature in this space remains nascent, several pilot studies have explored the potential role of DCE-MRI to improve patient stratification and therapy planning. A recent study conducted on 25 patients who underwent TARE utilizing ^90^Y resin or glass microspheres compared the use of DCE-MRI with Tc-99m MAA SPECT scans to estimate lung shunt fraction (LSF), a key parameter in treatment planning. Elevated intrahepatic arteriovenous shunting can divert microspheres away from the tumor and into the lungs, increasing the risk of radiation pneumonitis [53]. In addition, lung shunting may reduce the delivered dose to the tumor, compromising treatment efficacy [54]. The study found a significant correlation between the velocity |***u***| of blood flow obtained through quantitative transport mapping (QTM) and the LSF derived from SPECT post Tc-99m administration [33]. This approach capitalizes on DCE-MRI’s ability to capture changes in tumor blood flow and vasculature, correlating significantly with LSF and potentially eliminating the need for additional mapping procedures. Beyond perfusion assessment to predict bead distribution, pre-treatment DCE-MRI parameters have shown potential for the stratification of patients who are most likely to respond to radioembolization. A study evaluating histogram-based DCE-MRI tissue perfusion parameters in 25 patients at 6 weeks and 6–12 months post-TARE assessed arterial flow (*F_a_*), portal venous flow (*F_p_*), total flow (*F_t_*), arterial fraction, mean transit time, interstitial volume (*v_e_*), intracellular uptake rate (*K_i_*), and uptake fraction (*f_i_*) [32]. The analysis found that tumors with low heterogeneity prior to treatment, as measured by standard deviation and skewness, were more likely to achieve complete response to low-dose TARE within 6 weeks. These findings suggest that baseline histogram analysis of DCE-MRI data could be useful for stratifying patients more likely to respond to radioembolization.

By capturing spatial differences in vascularity, DCE-MRI has the potential to inform predictions of microsphere distribution and improve dose targeting. Preliminary studies have also indicated that baseline perfusion metrics may correlate with treatment response, raising the possibility that DCE-MRI could one day contribute to patient stratification, personalized dosimetry, and response monitoring. However, these findings remain exploratory, and larger, prospective studies are needed to validate the technique’s feasibility and clinical utility. Future work should focus on expanding patient cohorts and directly comparing DCE-MRI with existing dosimetry approaches to validate its potential in guiding personalized TARE strategies.

## 6. Limitations in DCE-MRI

Leveraging DCE-MRI for the purposes of guiding locoregional therapies presents several challenges and limitations. One major hurdle is the technical complexity of acquiring and interpreting DCE-MRI data. Achieving high temporal resolution is critical for capturing the dynamics of contrast agent distribution for quantitative analysis, as insufficient temporal resolution can lead to the underestimation of K^trans^ and k_ep_ and the overestimation of v_p_ [55]. However, achieving high temporal resolution often requires a trade-off with spatial resolution [55]. In addition, breathing motion during dynamic, time-series data acquisition can introduce motion artifacts that limit the precision of calculated parameters during DCE-MRI. Image registration to correct for the liver motion is challenging, as pixel intensity variations can occur during both spatial displacement and contrast agent uptake, complicating voxel-based analysis [56]. Coordinating image acquisition with the timing of breath holds during this narrow temporal window can be difficult, potentially compromising image quality and the consistency of perfusion measurements [57].

Beyond data acquisition, deriving clinically relevant quantitative perfusion parameters adds another layer of complexity, as the PK models used to interpret DCE-MRI data may not perfectly fit the observed patterns. This is because the models make assumptions that may not be completely physiologically valid, such as the assumption of uniform dispersion of the arterial input throughout an organ [58]. Additionally, due to the discrete nature of data acquisition, gaps in the time–intensity curves may lead to the loss of critical details in the arterial input function, potentially affecting the accuracy of perfusion measurements [59].

Further complicating the clinical translation of DCE-MRI is the variability in the quantitative imaging biomarkers derived from DCE-MRI. The process of obtaining PK parameters involves multiple steps in data acquisition and analysis, introducing inconsistencies due to differences in imaging protocols, scanner platforms, computational algorithms, and software tools. These inconsistencies can compromise the accuracy and reproducibility of derived PK parameters, ultimately reducing their predictive value and increasing the sample sizes required for clinical trials to achieve statistical significance.

For the clinical adoption of DCE-MRI, these challenges must be addressed by improving image acquisition approaches to increase temporal resolution without compromising spatial resolution, image registration approaches to align the images acquired at different time points, and consistent modeling approaches to compare the effectiveness of various PK models.

## 7. Limitations in Clinical Translation

The implementation of DCE-MRI into routine clinical workflows for guiding locoregional therapy faces several logistical and practical hurdles. Currently, pre-procedural DCE-MRI is not a standard part of treatment planning for HCC, and imaging data derived from such scans are not routinely used to inform procedural decisions. This is due in part to limited protocol standardization [60], longer acquisition and processing times [61], and the need for expertise in pharmacokinetic modeling and interpretation. Additionally, although promising, its clinical value remains under investigation, which contributes significantly to its limited integration into standard treatment planning for HCC [62]. Such factors can pose barriers to integration. In addition to workflow hurdles, several biological barriers hinder widespread adoption. HCC arises in the context of underlying liver disease, which varies significantly across patients in terms of cirrhosis severity, vascular architecture, and hepatic function [63]. These factors influence contrast kinetics and complicate the interpretation of perfusion parameters. Moreover, the tumor biology of HCC is heterogeneous, and differences in vascularity, differentiation, and microenvironment may affect DCE-MRI measurements and their predictive value [64]. These patient- and tumor-level variables challenge the generalizability of findings derived from small or highly selected study populations.

To date, the use of DCE-MRI in HCC has largely been confined to research settings, particularly in early-phase clinical trials evaluating anti-angiogenic therapies, where it has served as a biomarker of tumor vascular response [65,66]. However, its application in guiding locoregional therapies remains relatively underexplored, with most studies focusing on feasibility assessments rather than clinical implementation. Furthermore, a large portion of research in this area is preclinical. While preclinical findings are promising, their clinical relevance is constrained by the difficulty in translating results from small models to larger patient populations. In addition, there are currently no high-level clinical studies demonstrating an increase in median overall survival with the utilization of DCE-MRI to monitor locoregional therapies such as ablation. This limitation is primarily due to the challenges associated with conducting randomized controlled trials in interventional radiology. Additionally, the lack of pre-procedure imaging further limits the clinical implementation of DCE-MRI.

As the field progresses, it will be crucial to address the logistical and biological barriers to integration. Larger, multicenter clinical trials with more diverse patient cohorts are needed to establish the clinical validity and reproducibility of DCE-MRI across various stages and subtypes of HCC. Protocol standardization in acquisition, post-processing, and pharmacokinetic modeling will be crucial to widespread implementation. Given the heterogeneity of HCC tumor biology and pathological features, future studies should seek to integrate DCE-MRI data with other clinical and imaging biomarkers, such as liver function scores, radiomics, or genomic markers, to refine patient selection and personalize treatment plans. Ultimately, demonstrating that quantitative DCE-MRI can enhance the accuracy of treatment response assessment and improve patient outcomes will be fundamental in securing its position as a standard tool in guiding locoregional therapies for HCC.

## 8. Conclusions

In conclusion, DCE-MRI presents a promising imaging technique for quantifying liver and tumor perfusion, with the potential to refine treatment strategies and improve patient outcomes in the future. However, realizing this significant potential is contingent upon addressing significant challenges that currently hinder its widespread clinical adoption. These primarily involve standardizing imaging protocols and validating quantitative models to interpret the complex perfusion patterns observed in HCC tumors accurately.

In the context of ablation therapies, DCE-MRI could offer valuable insights into tumor microvascular characteristics, potentially guiding energy delivery during thermal ablation. For TACE, quantitative DCE-MRI parameters such as K^trans^ may serve as biomarkers for assessing treatment response and predicting tumor recurrence, especially when combined with molecular-targeted therapies. For ^90^Y TARE, quantitative DCE-MRI could improve dosimetry planning by providing more accurate assessments of tumor vascularity and microsphere distribution.

While these possibilities are promising, it is clear that quantitative DCE-MRI is not yet a routine part of clinical practice or has established guidelines for guiding locoregional therapies. Consequently, a concerted effort in further research and validation is needed before it can be reliably integrated into treatment planning. This essential next phase of research should prioritize large-scale prospective multicenter trials to confirm preliminary efficacy, collaborative efforts for technical standardization, and studies directly comparing DCE-MRI guided strategies with current standards of care.

If these challenges are successfully navigated, DCE-MRI could become a pivotal tool in personalizing treatment strategies for HCC, ultimately leading to improved patient care and potentially, enhanced survival outcomes.

## Figures and Tables

**Table 1:** Definition of semiquantitative and quantitative metrics of DCE-MRI.

Model	DCE-MRI Parameter Symbol	Units	Definition
Semiquantitative	Onset Time	s	Time of contrast agent onset
Semiquantitative	TTP	s	Time to maximum contrast enhancement
Semiquantitative	Peak Enhancement	SI	Maximum signal intensity reached within a tissue region after contrast injection
Semiquantitative	Peak Enhancement Ratio	%	Peak enhancement relative to the signal intensity at time of onset
Semiquantitative	Wash-in Slope	%	Speed of contrast uptake
Semiquantitative	Wash-out Slope	%	Speed of contrast clearance
Semiquantitative	AUC		Area under the signal intensity-time curve
Quantitative	K^trans^	min^−1^	Forward volume transfer coefficient between blood plasma and EES
Quantitative	k_ep_	min^−1^	Reverse volume transfer coefficient between blood plasma and EES
Quantitative	v_e_	%	EES volume per unit volume of tissue
Quantitative	v_p_	%	Blood plasma volume per unit volume of tissue
Quantitative	DV	%	Distribution volume of contrast agent in the total volume of tissue
Quantitative	F	mL·g^−1^·min^−1^	Total hepatic blood flow
Quantitative	F_a_	mL·g^−1^·min^−1^	Hepatic artery blood flow
Quantitative	F_p_	mL·g^−1^·min^−1^	Hepatic portal blood flow
Quantitative	MTT	s	Average time for plasma to traverse from arterial to the venous end of the vasculature

Note—TTP = time to peak; SI = signal intensity; AUC = area under the curve; DV = distribution volume; F = total liver blood flow; F_a_ = arterial–venous blood flow; F_p_ = portal venous blood flow; MTT = mean transit time; EES = extracellular extravascular space.

**Table 2:** Summary of studies that integrate dynamic contrast-enhanced MRI methods with locoregional therapies for hepatocellular carcinoma.

Study and Year	Treatment Method	Subject	Sample Size	Model	Valuable DCE Parameters	Major Results
Mostafa, 2016; [22]	RFA	Humans	50	Qualitative	Signal intensity, heterogeneity, pattern of enhancement, border definition	Dynamic contrast-enhanced MRI (DCE-MRI) showed early arterial enhancement and rapid wash-out in patients with recurrent and residual lesions at 1- and 3-month follow-ups.
Chiang et al., 2023; [23]	MWA	Porcine	5	Quantitative	K^trans^, k_ep_	Perfusion parameters were significantly lower in embolized liver lobes than in nonembolized liver lobes, and there was a moderate but significant correlation between normalized k_ep_ and ablation volume.
Moon et al., 2016; [24]	RFA	Rabbit	9	Quantitative	K^trans^, v_e_, v_p_	Measuring from both the partial necrotic area (PNA) and viable tumor area (VTA), mean K^trans^ values were positively correlated with mean microvascular density (MVD).
Ren et al., 2024; [25]	MWA, donafenib	Rabbit	40	Quantitative	K^trans^	K^trans^ and tumor diameter were significantly greater in the insufficient MWA group than in the control sufficient MWA group. The serum vascular endothelial growth factor (VEGF) concentration, K^trans^, and tumor diameter were significantly lower in the combined treatment group than in the other two groups.
Saleh et al., 2019; [26]	TACE	Humans	30	Qualitative	Arterial-phase hyperenhancement	DCE-MRI showed a 100% level of sensitivity using the application of the LI-RADS v2018 (the Liver Imaging Reporting and Data System) diagnostic algorithmic approach to evaluate HCC viability following TACE.
Svecic et al., 2021; [27]	TACE	Humans	366	Quantitative	Spatial–temporal features of DCE-MRI images pre-TACE, Onset time (To), time to peak (TTP), peak enhancement (ΔS), peak enhancement ratio (PER), normalized maximum intensity time ratio (nMITR), wash-in slope, wash-out slope, distribution volume (DV), arterial fraction (ART), K_2_, K_a_, K_p_	The Spatial–Temporal Discriminant Graph Neural Network predicted post-TACE response with 93.5% accuracy and generated follow-up images with no significant differences in perfusion parameters compared to ground-truth post-TACE examinations.
Kuang et al., 2021; [28]	TACE	Humans	153	Quantitative	DCE-MRI arterial phase features pre-TACE	Nomograms combining DCE-MRI arterial phase radiomics features with clinical variables predicted short-term response in HCC ≤ 5 cm with AUC = 0.83 (training) and 0.81 (validation), outperforming radiomics-only and clinical-only models.
Thibodeau-Antonacci et al, 2019; [29]	TACE	Humans	28	Semiquantitative	Onset time (To), time to peak (TTP), peak enhancement (ΔS), peak enhancement ratio (PER), normalized maximum intensity time ratio (nMITR), wash-in slope, wash-out slope	For non-viable tumors, time to peak increased after TACE. For equivocal or viable tumors, time to peak and mean transit time significantly increased and the transfer constant from the extracellular, and extravascular space to the central vein significantly decreased.
Saito et al., 2018; [30]	TACE, Sorafenib	Humans	11	Quantitative	Distribution volume of contrast agent (DV), K^trans^	DV was reduced in responders at 3 and 10 days post-TACE. DV fell in non-responders at three days but was not significantly changed from pre-TACE values after sorafenib.
Pokuri et al., 2018; [31]	TACE, Sunitinib	Humans	16	Quantitative	K^trans^	Mean K^trans^ and viable tumor percent decreased with combination therapy.
Hectors et al., 2020; [32]	^90^Y	Humans	24	Semiquantitative/Quantitative	Arterial–venous blood flow, portal venous blood flow, total liver blood flow, arterial flow fraction, mean transit time, v_e_, intracellular uptake rate (Ki), uptake fraction (fi)	Tumor DCE-MRI parameters of arterial–venous blood flow, arterial fraction, and v_e_ showed a significant reduction at 6 weeks post radioembolization compared with baseline values, whereas fi showed a significant increase.
Zhang et al., 2022; [33]	^90^Y	Humans	25	Quantitative	K^trans^, v_e_, Quantitative transport mapping (QTM) velocity	DCE-MRI with quantitative transport mapping demonstrated significant correlation between QTM velocity (|***u***|) and lung shunting fraction (LSF), along with increased K^trans^ and v_e_ in the high LSF group.

Note—RFA = radiofrequency ablation; MWA = microwave ablation; TACE = transarterial chemoembolization; K^trans^ = forward volume transfer coefficient between blood plasma and extracellular extravascular space (EES); k_ep_ = reverse volume transfer coefficient between blood plasma and EES; v_e_ = EES volume per unit volume of tissue; v_p_ = blood plasma volume per unit volume of tissue; K_2_ = transfer constant from the liver tissue to the central vein; K_a_ = transfer constant from the arterial plasma to EES; K_p_ = transfer constant from the portal venous plasma to the surrounding tissue.

## Data Availability

Data sharing does not apply to this article as no new data were created or analyzed in this study.

## Abbreviations

The following abbreviations are used in this manuscript:

DCE-MRI	Dynamic Contrast-Enhanced Magnetic Resonance Imaging
LRT	Locoregional Therapies
HCC	Hepatocellular Carcinoma
RFA	Radiofrequency Ablation
MWA	Microwave Ablation
TACE	Transarterial Chemoembolization
TARE	Transarterial Radioembolization
BCLC	Barcelona Clinic Liver Cancer
CE-MRI	Contrast-Enhanced Magnetic Resonance Imaging
PK	Pharmacokinetic
